# Targeting the TR4 nuclear receptor with antagonist bexarotene can suppress the proopiomelanocortin signalling in AtT‐20 cells

**DOI:** 10.1111/jcmm.16074

**Published:** 2021-01-24

**Authors:** Liqun Xia, Danyang Shen, Youyun Zhang, Jieyang Lu, Mingchao Wang, Huan Wang, Yuanlei Chen, Dingwei Xue, Dajiang Xie, Gonghui Li

**Affiliations:** ^1^ Department of Urology Sir Run Run Shaw Hospital Zhejiang University School of Medicine Hangzhou China; ^2^ Department of Neurosurgery Sir Run Run Shaw Hospital Zhejiang University School of Medicine Hangzhou China

**Keywords:** cortisol, Cushing's disease, pituitary tumours, proopiomelanocortin, testicular receptor 4

## Abstract

Drug options for the life‐threatening Cushing's disease are limited, and surgical resection or radiation therapy is not invariably effective. Testicular receptor 4 (TR4) has been identified as a novel drug target to treat Cushing's disease. We built the structure model of TR4 and searched the TR4 antagonist candidate via in silico virtual screening. Bexarotene was identified as an antagonist of TR4 that can directly interact with TR4 ligand binding domain (TR4‐LBD) and induces a conformational change in the secondary structure of TR4‐LBD. Bexarotene suppressed AtT‐20 cell growth, proopiomelanocortin (POMC) expression and adrenocorticotropin (ACTH) secretion. Mechanism dissection revealed that bexarotene could suppress TR4‐increased POMC expression *via* promoting the TR4 translocation from the nucleus to the cytoplasm. This TR4 translocation might then result in reducing the TR4 binding to the TR4 response element (TR4RE) on the 5’ promoter region of POMC. Results from in vivo mouse model also revealed that oral bexarotene administration markedly suppressed ACTH‐secreting tumour growth, adrenal enlargement and the secretion of ACTH and corticosterone in mice with already established tumours. Together, these results suggest that bexarotene may be developed as a potential novel therapeutic drug to better suppress Cushing's disease.

## 1 INTRODUCTION

Cushing's disease (CD) is a neuroendocrine disorder caused by the excessive production of adrenocorticotropin (ACTH) by the pituitary gland resulting in hypercortisolism. Though the prevalence of CD is low (~4/10^5^), patients with CD show a mortality rate that is four times higher than that of the general population due to metabolic, cardiovascular, psychiatric disorders or infections induced by high levels of cortisol exposure.^1^ The first‐line treatment for CD involves the surgical resection of the underlying pituitary adenomas, which is associated with a complete remission rate of 60%–90% for microadenomas and <65% for macroadenomas.^2^ The postsurgical persistence of ACTH hypersecretion or residual tumours may turn to radiotherapy, yet its therapeutic effects may take years to be achieved and are associated with risk of pituitary damage.^3^ Several drugs have been approved by regulatory authorities to treat CD, including pituitary‐targeted pasireotide, adrenal‐targeted ketoconazole, and glucocorticoid receptor antagonist mifepristone. Metyrapone, etomidate, mitotane and cabergoline are off‐label drugs for CD treatment.^4^ However, medical therapy is generally adjunctive with limited use because of its side effects. Thus, the development of new drugs for CD treatment is extremely urgent especially for patients who have low tolerance for surgery and radiotherapy.

The testicular receptor 4 (TR4) is a member of the nuclear receptor superfamily. Studies from TR4 knocking out mice reveal that TR4 plays essential roles in many normal physiological processes, such as fertility,^5^, ^6^ glucose ^7^ and lipid metabolism,^8^ oxidative stress,^9^, ^10^ erythroid differentiation,^11^ and bone metabolism.^12^ Moreover, TR4 affects the tumour progression and therapy resistance of several cancers. TR4 promotes the metastasis of prostate cancer,^13^, ^14^, ^15^ renal cell carcinoma,^16^ and seminoma ^17^ but inhibits the progression of hepatic cell carcinoma.^18^ Recently, Du et al found that TR4 promotes the phenotype of CD, including the cell growth of pituitary adenoma as well as its secretion of ACTH and the followed corticosterone secretion.^19^ A mechanism study revealed that TR4 can bind to the promoter region of proopiomelanocortin (POMC), an ACTH precursor polypeptide, and thus transcriptional regulate the expression of POMC.^19^ Zhang et al showed that TR4 may form heterodimers with the glucocorticoid receptor (GR) and thus block the transcriptional inhibition of GR on POMC.^20^ Hence, TR4 could serve as a potential target for CD treatment. However, a little progress has been made on the development of TR4 inhibitors. High concentrations of metformin (500 µmol/L) could inhibit TR4 transactivation through the induction of TR4 phosphorylation at site Ser351 in hepatocytes.^7^ Lately, a selective MEK1/2 inhibitor, MEK‐162, demonstrated its potential for treating CD through the suppression of ERK1/2‐mediated phosphorylation activation of TR4,^21^ which provided an example to treat CD by targeting TR4.

Bexarotene (also known as Targretin, LGD1069) is a second‐generation retinoic X receptors (RXRs) agonist, which has been approved by the FDA to treat all stages of cutaneous T cell lymphoma (CTCL) since 1999.^22^ After long‐term bexarotene treatment, some CTCL patients may experience reversible hypopituitarism accompanied by decreased plasma ACTH and cortisol.^23^, ^24^ This observation indicates that bexarotene may act on the pituitary gland to treat CD. However, the therapeutic effect and underlying mechanisms of bexarotene on CD are still elusive.

In this study, we identified bexarotene as an inhibitor of TR4. Bexarotene physically binds to TR4‐LBD and subsequently induces a conformation change, as indicated by the surface plasmon resonance (SPR) and circular dichroism assay. Then, we validated the capacity of bexarotene to treat CD. Bexarotene could effectively suppress the cell growth, POMC expression, and ACTH secretion of AtT‐20 cells. Moreover, bexarotene treatment led to a distinct abrogation of pituitary tumours as well as a significant reduction of plasma ACTH and cortisol.

## 2 MATERIALS AND METHODS

### 2.1 Molecular modelling and docking analysis

The receptor model of the TR4‐LBD domain in active confirmation was built on the crystal structure of RXR‐alpha (PDB: 4K6I) by using Modeller.^25^ Water molecules, ligands and other hetero atoms were removed from the protein molecule. The missing hydrogen atoms of the proteins were added, and the energy minimization of protein was performed to remove bumps and correct the covalent geometry. The molecular structures of the ligand were downloaded from the Zinc15 database.^26^ Thereafter, virtual screening was performed with AutoDock Vina under the default docking parameters,^27^ and point charges were initially assigned according to the AMBER03 force field.^28^

### 2.2 TR4‐LBD expression and purification

The TR4‐LBD prokaryotic expression plasmid (pSumo‐TR4‐LBD) was provided by Dr Zhou from the University of Michigan Medical School. TR4‐LBD was heterologously expressed in BL21 (DE3). The resulting proteins that were tagged with a His_6_‐sumo‐motif at their N‐termini were isolated and purified by using cOmplete His‐tag purification column (Roche, Mannheim, Germany). His‐sumo‐motif was then removed by using the RobustCutter Sumo protease (robustnique, Tianjin, China). Another cOmplete His‐tag purification was applied to collect the TR4‐LBD protein in the flowthrough. Protein concentrations were determined by the BCA method (Thermo Fisher Scientific).

### 2.3 Surface Plasmon resonance

Binding experiments were performed with the surface plasmon resonance (SPR)–based biosensor instrument PlexArray^®^ HT (Plexera Bioscience). The FDA‐approved drug library, which includes bexarotene and All‐Trans Retinoic Acid (ATRA), were immobilized on the sensor surface according to the standard procedure and manufacturer's instructions. The buffer of the TR4‐LBD protein was changed to the SPR buffer (20 mmol/L HEPES, 150 mmol/L NaCl, pH7.5) by using the HiTrap desalting column (GE Healthcare, Stockholm, Sweden). Variable concentrations of TR4‐LBD were injected at 2 μL/s, and binding to the small molecules immobilized on the chip was monitored in real time. Each sensorgram consists of an association phase (300 seconds), reflecting the binding of the injected protein to the drugs followed by a dissociation phase (300 seconds), during which the running buffer was passed over the chip and the bound TR4‐LBD was washed off the drug surface.

### 2.4 Circular dichroism spectrum analysis

Circular dichroism measurements were conducted with a Jasco J‐815 spectropolarimeter (JASCO, Tokyo, Japan). The circular dichroism spectra of 5 mmol/L phosphate buffer (pH 7.4) were obtained by using a cell with a 0.5 cm path length. TR4‐LBD protein at a concentration of 0.2 mg/mL with or without bexarotene (20 μmol/L) was measured with a spectropolarimeter.

### 2.5 Cell lines and reagents

The mouse anterior pituitary corticotroph adenoma cell line AtT‐20/D16v‐F2 was purchased from ATCC, while COS‐7 cell line was purchased from Cell Bank of Type Culture Collection of Chinese Academy of Sciences. AtT‐20 cells and COS‐7 cells were cultured in DMEM (ATCC, 30‐2002) media supplemented with 10% foetal bovine serum (Cellmax, Peking, China) and penicillin‐streptomycin. The cells were incubated at 37°C and 5% CO_2_ atmosphere. Bexarotene, ATRA, and dexamethasone (DEX) were obtained from Selleck and stored at −80°C. Corticotropin‐releasing factor human (CRH) was obtained from MCE and stored at −80°C.

### 2.6 MTT assay

MTT was used for counting cell numbers. AtT‐20 cells (1 × 10^4^ cells/well) seeded in 96‐well plates were incubated at regular medium for 24 hours. Then, the cells were treated with bexarotene (1‐10 μmol/L) for 72 hours in 2% FBS medium. Afterwards, one‐tenth volume of MTT was added to each well, and the plate was continuously incubated for another 2 hours at 37°C and 5% CO_2_ atmosphere. The absorbance was measured at 550 nm with a microplate reader (Bio‐Tek).

### 2.7 EdU incorporation assay

An EdU Cell Proliferation Assay Kit (Solarbio, America) was applied to determine the effect of bexarotene on cell proliferation. Cells were cultured in triplicate in 24‐well plates at a density of 2 × 10^5^ and treated with bexarotene (0, 5, 10 μmol/L) for 24 hours in 2% FBS medium. Then 50 μmol/L EdU was added to each well and cells were cultured for another 2 hours. Afterwards, the cells were fixed, permeabilized and stained according to the manufacturer's instruction. The result was visualized using a fluorescent microscope (Axio Observer A1, Germany).

### 2.8 Real‐time–qPCR (RT–qPCR)

AtT‐20 cells grown to 60%–70% confluence in regular medium in 6‐well plates were incubated with bexarotene in 2% charcoal‐stripped FBS medium for 12, 24, or 48 hours. Total RNA was extracted with TRIzol (Cwbiotech, Peking, China) according to the manufacturer's instructions. cDNA synthesis was performed by using All‐in‐One cDNA Synthesis SuperMix (Bimake, Shanghai, China). RT‐PCR reactions were performed in 2 × SYBR Green qPCR master mix (Bimake, Shanghai, China). The primer sequences are available in Table S1. The relative expression was calculated using the comparative ΔΔCt method.

### 2.9 Western blot analysis

AtT‐20 cells that were grown to 60%–70% confluence in regular medium in 6‐well plates were incubated with bexarotene in 2% charcoal‐stripped FBS medium for 24 hours. Then, the cells were washed with cold PBS and lysed in cell lysis buffer (Beyotime, Shanghai, China) with protease inhibitor cocktail (Bimake, Shanghai, China) on ice for 10 minutes. Then, the lysates were collected and centrifuged at 12 000 *g* for 20 minutes. Protein concentrations were quantitated by using a BCA protein assay kit (Thermo Fisher), and 20 µg of total proteins were separated on the SDS/PAGE gels, which were then transferred to polyvinylidene difluoride membranes. Blots were probed with GAPDH (ab181602, abcam), TR4 (ab109301, abcam), POMC (ab32893, abcam), PARP (ab227244, abcam), C‐caspase‐3 (ab2302, abcam), cyclin D1 (#2978, CST), CDK1 (#9116, CST), cyclin B1 (#4138, CST), cyclin A2 (#4656, CST) primary antibodies. Horseradish peroxidase (HRP)‐linked anti‐rabbit IgG and anti‐goat IgG secondary antibodies were incubated against each primary antibody, and the hybridization signals were detected by using an enhanced chemiluminescence detection kit (Fdbio‐tech).

### 2.10 Plasmids transfection and reporter assays

Upon reaching 60%–70% confluence in 48‐well plates, the AtT‐20 cells were transfected with 250 ng pGL3‐POMC‐Luc plasmid containing the −1800 bp rat POMC promoter‐luciferase reporter (RiboBio), 150 ng PRL‐TK(Δ238) renilla control plasmid, 100 ng pcDNA3.1/TR4 or pcDNA3.1 vector, and 100 ng TR4 shRNA or scramble control by using Lipofectamine 3000 (Invitrogen). After 24 hours of transfection, the medium was replaced with 2% charcoal‐stripped FBS medium, and the cells were treated with bexarotene for additional 24 hours. The siRNAs of EGFR, RARs and RXRs are listed in Table S2. Luciferase activity was measured with a dual‐luciferase assay kit (Promega) according to the manufacturer's instructions.

### 2.11 Immunofluorescence

The AtT‐20 cells were exposed to bexarotene in 2% charcoal‐stripped FBS medium for 24 hours. Then, the cells were fixed with 4% paraformaldehyde, incubated with 0.2% Triton‐X, and subsequently blocked with 5% BSA for 1 hour. The cells were incubated with rabbit anti‐TR4 primary antibody at 4°C overnight, and then incubated with Alexa Fluor 488 Goat anti‐Rabbit IgG3 cross‐adsorbed secondary antibody (Invitrogen) for 1 hour at room temperature. The nuclei were stained by DAPI for 15 minutes.

### 2.12 Cell cycle

The AtT‐20 cells were plated in 6‐well plates overnight and treated with bexarotene in 2% charcoal‐stripped FBS medium for 24 hours. The cell cycle profile was analysed with a cell cycle staining kit (Multi Science) according to the manufacturer's instructions. Cell cycle analysis was performed by using FACSCalibur (BD, NJ, USA).

### 2.13 Caspase activity measurement

The AtT‐20 cells were plated in 96‐well plates overnight and treated with bexarotene in a medium supplemented with 2% charcoal‐stripped FBS for 24 hours. Caspase‐3/7 activity was measured through the caspase cleavage of a luminogenic caspase‐3/7 substrate following the manufacturer's instructions (Promega Corporation).

### 2.14 ACTH electrochemiluminescent assay (ECLIA)

The AtT‐20 cells seeded in 12‐well plates were incubated with bexarotene, DEX or CRH, or their combinations in 1% charcoal‐stripped FBS medium for 24 hours. The supernatant was harvested. Then, the ACTH concentrations were measured through ECLIA (Roche Diagnostics) by using a Cobas E601 analyzer (Roche Diagnostics). The total protein in each well was extracted for the normalization of the data.

### 2.15 Chromatin immunoprecipitation (ChIP) assay

ChIP assay was performed according to the instructions in the SimpleChIP^®^ Enzymatic Chromatin IP kit. Briefly, the cells were cross‐linked with 1% formaldehyde and then quenched by adding glycine. The cells were resuspended in the ChIP sonication cell lysis buffer with protease inhibitor cocktail and then sonicated. After removing 5% of the solution for the evaluation of input complex, the lysates were immunoprecipitated overnight at 4°C by using 4 μg of TR4 antibody (or control IgG). Immunocomplexes were collected by using 30 μL of Protein G magnetic beads, and sequentially washed three times with low‐salt wash buffer and once with high‐salt buffer. DNA‐protein complexes were collected in the ChIP elution buffer and disrupted through incubation at 65°C for 30 minutes. The proteins were then removed through digestion with proteinase K at 65°C for 4 hours. The DNA fragments were purified by using a DNA purification kit. For PCR, the primer sequences were as follows: forward 5′–GTA GAT TAG GCA GGC ACC CCG ACT G–3′ and reverse 5′–GAA TGG TCT GGG TGG GGA TTG TCTG–3′.

### 2.16 Tumour xenografts and in vivo treatment

The AtT‐20 cells (8 × 10^5^) dissolved in PBS were inoculated subcutaneously in 6‐week‐old female nu/nu mice. After 2 weeks, the mice bearing palpable tumours were randomized to receive bexarotene (100 mg/kg/d) or vehicle for 18 days through intragastric administration. The body weights and tumour volumes were measured every 3 days. The mice were killed through CO_2_ inhalation. Their cardiac blood was collected, and tumours and adrenals were excised. Studies on animals were conducted with approval from the Animal Research Ethics Committee of Zhejiang University (IACUC: ZJU20170192).

### 2.17 H&E and immunohistochemical (IHC) staining

Tissues were fixed in 10% (v/v) formaldehyde in PBS, embedded in paraffin, and cut into 4 mm sections for H&E and IHC staining with rat POMC antibodies. After the antigen retrieval step, the slides were incubated with endogenous peroxidase blocking solution to inhibit endogenous peroxidase and then incubated with the POMC antibody, biotin‐conjugated secondary antibody, and enzyme conjugate HRP‐streptavidin. Freshly prepared DAB (Zymed, South San Francisco, CA) was used as substrate for the detection of HRP. Finally, the slides were counterstained with haematoxylin and mounted with aqueous mounting media.

### 2.18 Statistical analysis

Statistical analyses were performed using GraphPad Prism 6 (GraphPad Software, Inc). Data are presented as mean ± standard error of the mean (SEM) of three independent experiments, each performed at least in triplicate. Group differences were tested for statistical significance using Student's *t* test, one‐way ANOVA and two‐way ANOVA as appropriate. *P* < .05 was considered statistically significant.

## 3 RESULTS

### 3.1 Identification of bexarotene as an antagonist for TR4

**FIGURE 1:**
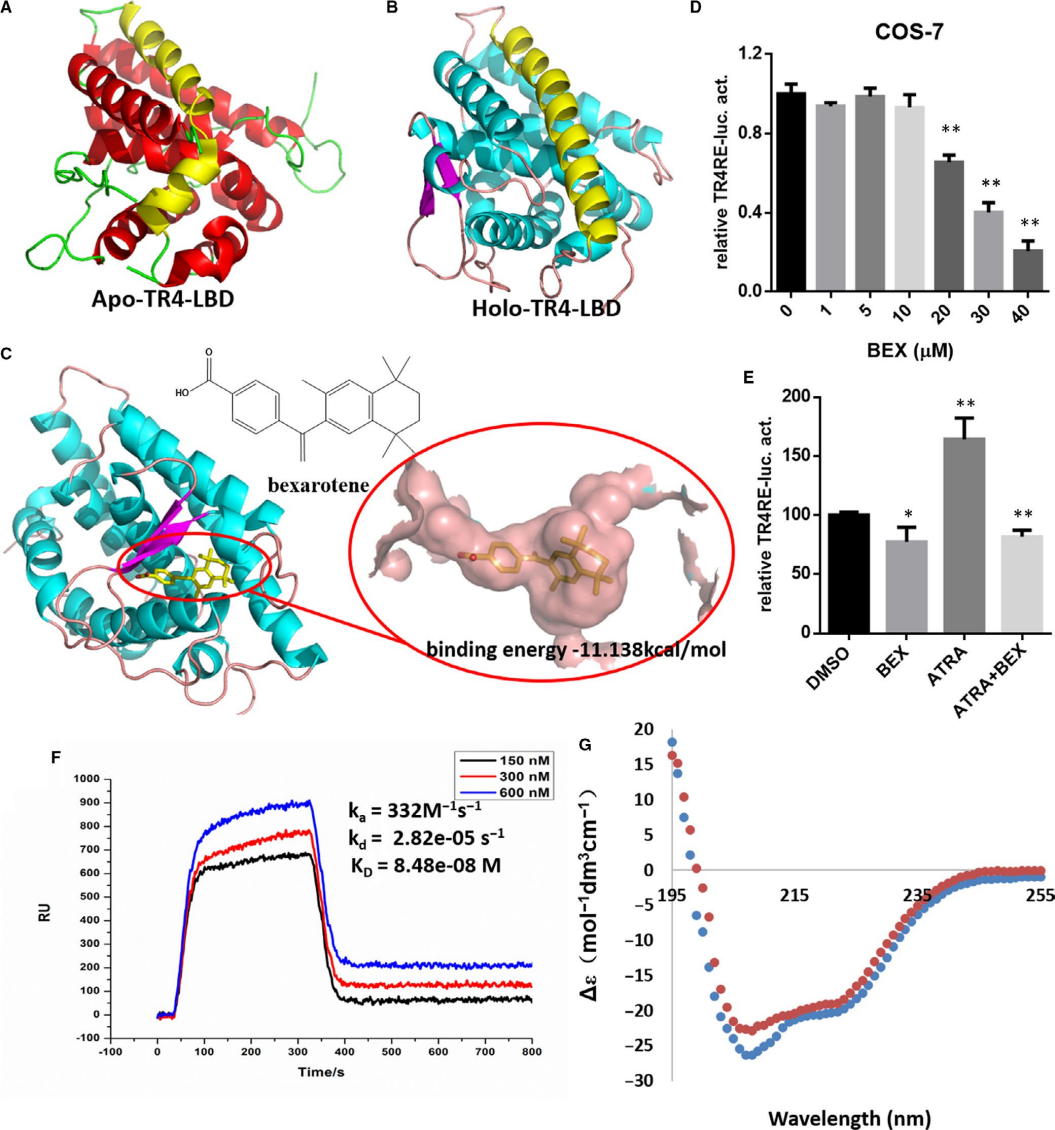
Identification of bexarotene as a TR4 inhibitor. A, 3D structure of TR4‐LBD, α‐helixes are in red, while loops are in green; B, Molecular model of TR4‐LBD with a ligand binding site, α‐helixes are in cyan, β‐sheets are in magenta, and loops are in salmon; the most significantly changed helix was marked in yellow; C, Molecular modelling of TR4‐LBD and its interaction with bexarotene in vitro; bexarotene is surrounded by hydrophobic loops; D, Bexarotene inhibited the TR4‐dependent transcriptional activity in COS‐7 cells as demonstrated by dual‐luciferase assay; E, Bexarotene inhibited the ATRA‐activated TR4 transactivation in COS‐7 cells as demonstrated by dual‐luciferase assay; F, Binding kinetic analysis of bexarotene to TR4–LBD by SPR; G, Analysis of bexarotene binding to TR4–LBD based on the circular dichroism spectrum. Data are the means ± SEM of three independent experiments. One‐way ANOVA was used to test differences for statistical significance. *P ˂ .05, **P ˂ .01

Virtual screening was applied for the identification of agonists/antagonists that can bind to TR4. The crystal structure of the TR4 ligand binding domain (TR4–LBD) was determined in 2011 (Figure 1A), yet the structure was in an auto‐inhibited inactive conformation, wherein the ligand‐binding pocket is occupied by the nearby helix.^29^ Sequence alignment shows that the sequence similarity of TR4 and RXRs reached ~50%. Thus, the model of TR4–LBD in active conformation was built according to the RXR‐alpha (PDB code: 4K6I).^30^ The constructed active TR4–LBD model (Figure 1B) was similar to the crystal structure of TR4–LBD (Figure 1A), except for the ligand‐binding region. We used this model to in silico search compounds that can bind to the ligand binding site from the ZINC database containing over 50 000 compounds. Compounds with extremely low binding energy underwent biological activity test. Of these compounds, bexarotene exhibited the highest inhibitory activity for TR4 transactivation (Figure 1C) and suppressed the TR4‐mediated transactivation in a dose‐dependent manner (Figure 1D). ATRA is a TR4 agonist.^29^ Bexarotene inhibited the transactivation of TR4 by ATRA (Figure 1E). These results demonstrate that bexarotene may serve as a TR4 antagonist.

To test the physical binding of bexarotene to TR4–LBD, we applied SPR to determine the binding affinity of bexarotene to TR4–LBD. The binding kinetics for bexarotene was estimated through the global fitting analysis of the titration curves to the 1:1 langmurian interaction model.^31^ The calculated *K*_D_ of bexarotene was 8.48 × 10^‐8^ mol/L according to the ratio of the dissociation rate constant (*k*_d_, 2.82 × 10^−5^ s^−1^) to the association rate constant (*k*_a_, 3.32 × 10^2^ mol/L s^−1^) (Figure 1F). ATRA showed a higher *K*_D_ (approximately 1 × 10^‐4^ mol/L) to TR4 than bexarotene, and this result is consistent with the functional results that bexarotene totally abolishes the activation of TR4 by ATRA (Figure S1). Furthermore, we performed circular dichroism spectroscopic assay to determine whether bexarotene induces a conformational change in TR4–LBD. The characteristic peak for alpha‐helix at 208 nm was significantly reduced (Figure 1G).

Together, results from Figure 1A‐G suggest that bexarotene can function as an antagonist of TR4 that can directly interact with TR4–LBD and induce a conformational change in the secondary structure of TR4–LBD.

### 3.2 Bexarotene inhibited the cell growth of ACTH‐secreting cells

**FIGURE 2:**
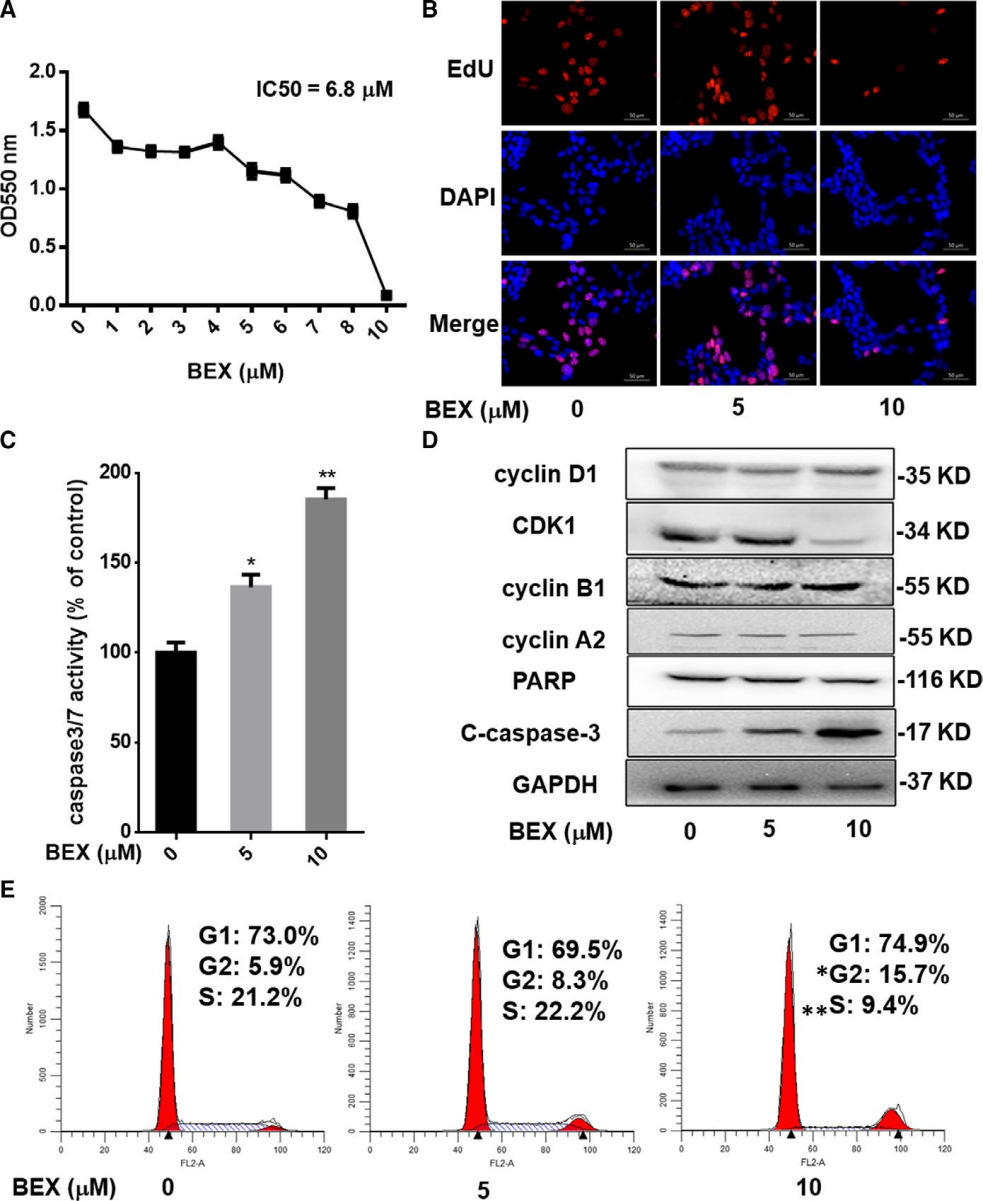
AtT‐20 cell growth as inhibited by bexarotene. A, Proliferation of AtT‐20 cells after 72 h of treatment with bexarotene was measured with MTT assay; B, EdU incorporation assay of AtT‐20 cells exposure to 0, 5 10 µmol/L bexarotene for 24 h; C, Bexarotene promoted AtT‐20 apoptosis as represented by elevated caspase3/7 activity; D, Apoptosis markers and cell cycle‐related proteins were detected by Western blot analysis; E, 24‐h bexarotene exposure induced G2/M phase arrest in AtT‐20 cells; data are the means ± SEM of three independent experiments. One‐way ANOVA was used to test differences for statistical significance. *P ˂ .05, **P ˂ .01

TR4 is a potential target of CD for its direct linkage to the aetiology of CD.^19^ Thus, we treated the murine corticotroph AtT‐20 cells with bexarotene to examine the potential function of the herein identified TR4 antagonist on CD in vitro. MTT assay revealed that bexarotene inhibited the cell proliferation of AtT‐20 in a dose‐dependent manner (Figure 2A). Similar results were also obtained when we replaced MTT assay with EdU incorporation assay, the number of proliferating cells decreased dramatically upon exposure to bexarotene (Figure 2B).

Furthermore, caspase 3/7 activity was visibly enhanced in AtT‐20 by bexarotene treatment (Figure 2C), suggesting the significant induction of apoptosis by bexarotene. The substantial increase in cleaved caspase‐3 further confirmed that bexarotene induced AtT‐20 cell apoptosis (Figure 2D). Cell cycle analysis revealed that bexarotene induced slight G2/M phase arrest, while the cell number of the G1 phase was similar to that of vehicle‐treated group (Figure 2E). Accordingly, the level of CDK1, which is involved in G2/M transition, was reduced upon exposure to bexarotene (Figure 2D).

Together, results from Figure 2A‐E suggest that TR4 antagonist bexarotene induced apoptosis and cell cycle arrest and thus inhibited ACTH‐secreting cell growth.

### 3.3 Bexarotene suppressed POMC expression and ACTH secretion

**FIGURE 3:**
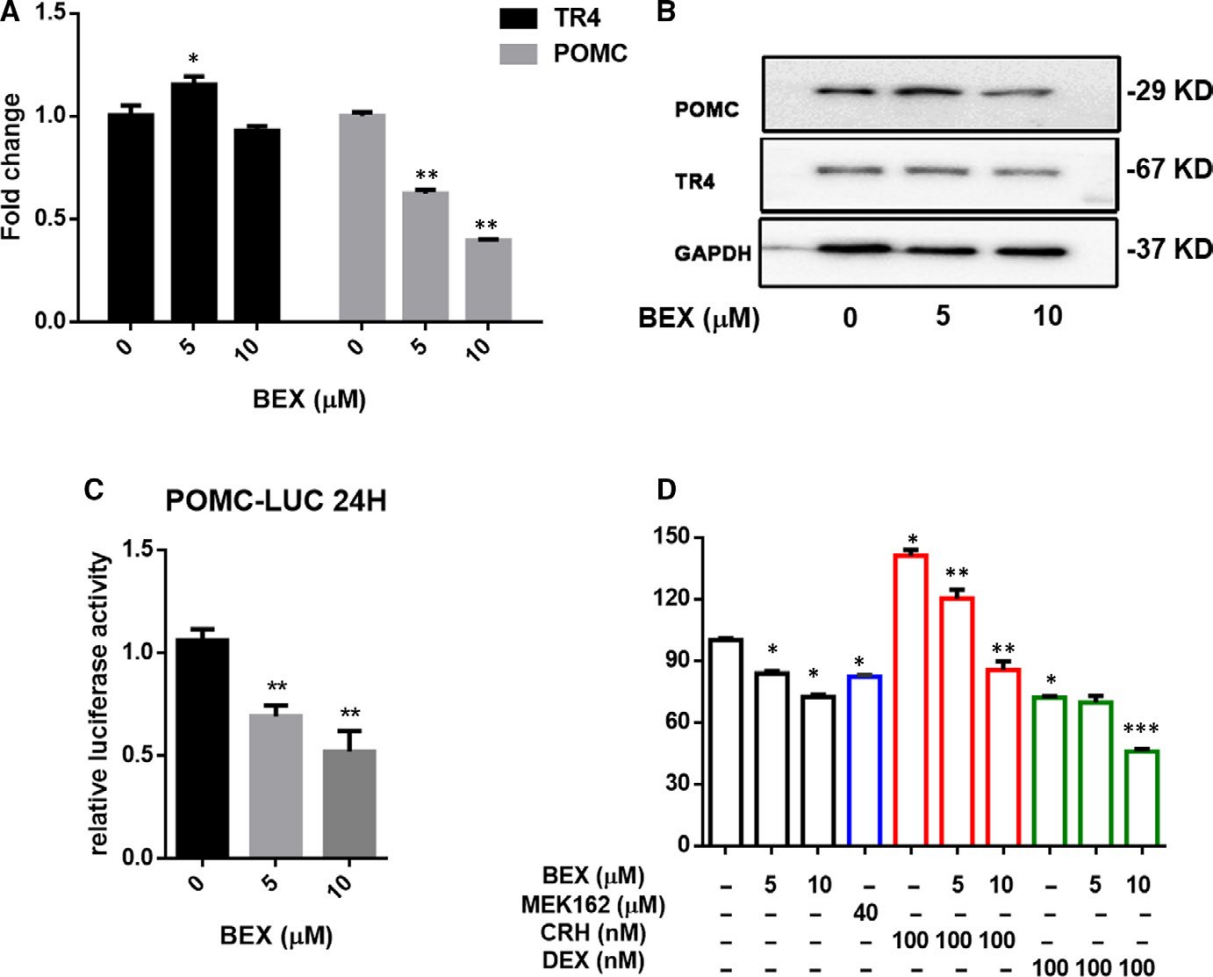
Bexarotene treatment reduces POMC transcription and ACTH secretion in AtT‐20 cells. A, Changes in POMC and TR4 mRNA after treatment with 0, 5 and 10 µmol/L bexarotene for 12 h were assessed by qPCR; B, Protein levels of POMC and TR4 after treatment with 0, 5 and 10 µmol/L bexarotene for 24 h were assessed by Western blot analysis. C, Cells were transfected with a POMC promoter‐luciferase reporter and pRL‐TK(Δ238); after 18 h, cells were treated with 0, 5 or 10 µmol/L bexarotene for 24 h, and luciferase activities were measured and presented as the fold change compared the control; D, AtT‐20 cells were treated for 24 h with 0, 5 or 10 µmol/L bexarotene, 40 µmol/L MEK‐162, 100 nmol/L CRH, 100 nmol/L DEX, or their combinations as indicated. ACTH levels were measured in the supernatants from AtT‐20 cells through ECLIA. Results are expressed as the percentages of each control. Data are the means * SEM of three independent experiments. *P ˂ .05, **P ˂ .01 except in Figure 4D *P ˂ .01, compared with the basal values, **P ˂ .01 compared with CRH stimulation, ***P ˂ .01 compared with DEX suppression. One‐way ANOVA and two‐way ANOVA were used to test differences for statistical significance

We first examined the effects of bexarotene on the regulation of POMC expression, as POMC is the precursor of ACTH which plays a key role in the progression of CD. Results from qPCR assay and Western blot analysis revealed that the expression of POMC in AtT‐20 cells was reduced after 12, 24, and 48 hours of bexarotene treatment at the mRNA level (Figure 3A and Figure S2), and the protein level (Figure 3B). In contrast, adding bexarotene resulted in little change of the TR4 protein expression, despite the fluctuation in TR4 mRNA expression (Figure 3A and Figure S2). We further studied the bexarotene regulation of POMC expression at the transcriptional level. Results from dual‐luciferase reporter assay revealed that bexarotene inhibited luciferase activity driven by the POMC promoter in a dose‐dependent manner (Figure 3C). Along with the changes in POMC, ACTH secretion was inhibited by bexarotene (Figure 3D), demonstrating a curative effect of bexarotene as a pituitary‐targeted drug. Notably, the inhibitory effect of 10 µmol/L bexarotene on ACTH secretion was superior to that of 40 µmol/L MEK162, a MEK1/2 inhibitor which is able to inhibit phosphorylation activation of TR4.^21^ Moreover, bexarotene inhibited the induction of ACTH secretion by CRH and promoted the inhibition of ACTH by DEX (Figure 3D).

Together, results from Figure 3A‐D suggest that bexarotene can effectively suppress POMC expression and ACTH secretion via reducing POMC transcription.

### 3.4 Inhibition of bexarotene on POMC transcription as mediated by TR4

**FIGURE 4:**
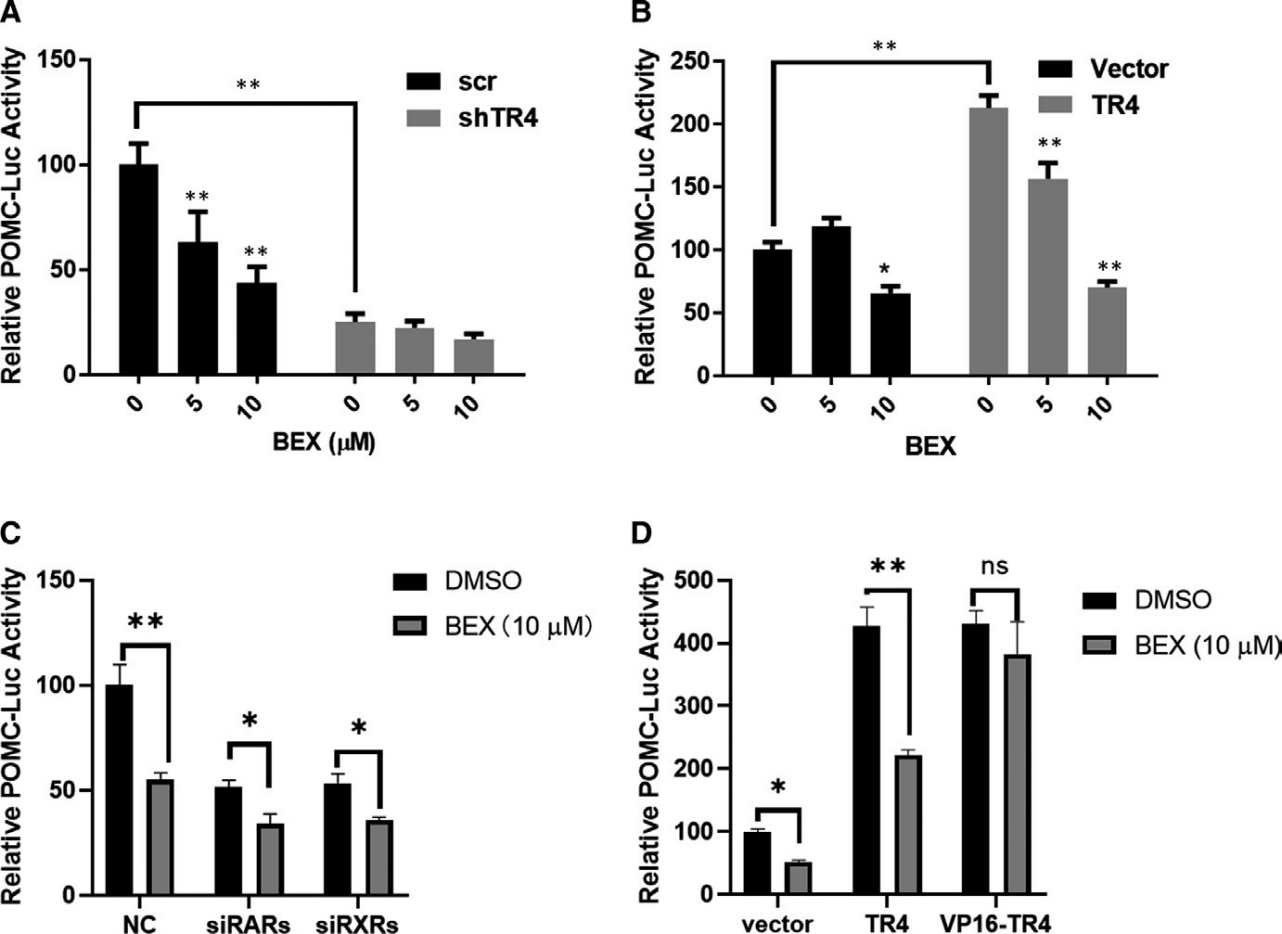
Bexarotene exerted its inhibitory effect through TR4. shTR4 or scramble plasmid (A), pcDNA3.1‐TR4 or vector plasmid (B) were co‐transfected with a POMC promoter‐luciferase reporter and pRL‐TK (Δ238). After 18 h, cells were treated with 0, 5 or 10 µmol/L bexarotene for 24 h, and luciferase activities were measured and presented as the fold change compared with the control. Cells were transfected with a POMC promoter‐luciferase reporter, pRL‐TK(Δ238) and siRNAs (C) or TR4 overexpression plasmid (D). After 18 h, cells were treated with 0 or 10 µmol/L bexarotene for 24 h, and luciferase activities were measured and presented as the fold change compared with the control. Data are the means ± SEM of three independent experiments. One‐way ANOVA and two‐way ANOVA were used to test differences for statistical significance. *P ˂ .05, **P ˂ .01

To further clarify the effect of bexarotene through the inhibition of TR4, we compared the POMC‐luciferase (POMC‐luc) activity of bexarotene treatment in the presence of different TR4 levels (Figure S3A‐C). Consistent with previous result, the knockdown of TR4 significantly suppressed the basal POMC‐luc activity, whereas the overexpression of TR4 promoted POMC‐luc activity in the AtT‐20 cell. Bexarotene inhibited the POMC‐luc activity in a dose‐dependent manner in the AtT‐20 cells transfected with a scramble control, whereas TR4 knockdown totally blocked the inhibitory effect of bexarotene on the POMC‐luc activity (Figure 4A). By contrast, the inhibitory effect of bexarotene on POMC‐luc activity was augmented in the TR4‐overexpressed AtT‐20 cells (Figure 4B). In addition, we found that down‐regulation of TR4 significantly reduced the cell growth of AtT‐20 cells and the growth inhibition rate of bexarotene was reduced in the shTR4 group compared to the scramble group (Figure S4A,B), indicating that bexarotene exerted its growth inhibition on AtT‐20 partially through TR4. Moreover, the ACTH secretion inhibition by bexarotene was almost blocked in the shTR4 group, suggesting the inhibition of POMC transcription by bexarotene via TR4 eventually lead to decreased ACTH secretion (Figure S4C).

As bexarotene is a well characterized RXRs agonist, we knocked down RXRs or RARs respectively to check whether bexarotene exerted its function through the activation of RAR/RXR signalling (Figure S3D,E). The results revealed that down‐regulation of RXRs or RARs could significantly suppress the POMC‐luc activity, however, it will not abolish the inhibitory effect of bexarotene on the POMC‐luc activity (Figure 4C). Moreover, we constructed a VP16 tagged TR4 overexpression plasmid (VP16‐TR4), this TR4 construct is not dependent on ligand‐induced conformational changes for nuclear localization or coactivator recruitment due to the strong transactivation and nuclear localization properties provided by the VP16 domain. As expected, VP16‐TR4 could induce POMC transcription to a similar extent to that of TR4, however, bexarotene could not inhibit the activation of POMC‐luc activity induced by VP16‐TR4 (Figure 4D). Hence, we concluded that bexarotene suppressed the transactivation activity of TR4 and reduced POMC transcription.

### 3.5 Mechanism dissection of how bexarotene can suppress TR4 function: *via* altering the nuclear‐cytoplasmic distribution of TR4

**FIGURE 5:**
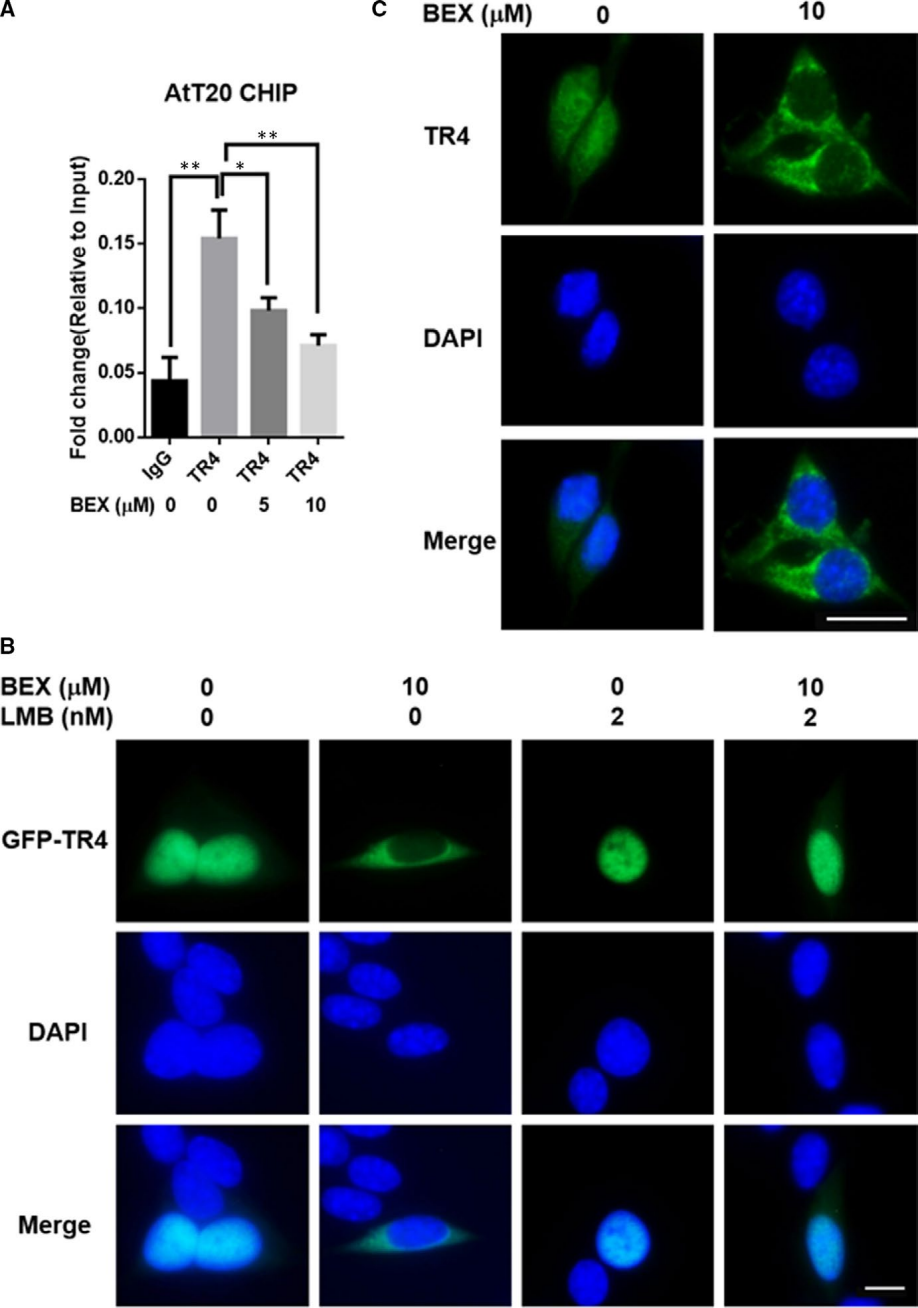
Bexarotene inhibited the nuclear translocation of TR4 to suppress its transactivation. A, AtT‐20 cells were treated with 0, 5 and 10 µmol/L bexarotene for 24 h, after which ChIP assay was performed to measure the TR4 binding to the POMC promoter (−854 bp~−637 bp). B, GFP‐TR4 was translocated to the cytoplasm upon bexarotene treatment. Pretreatment with LMB (1 ng/mL) for 3 h blocked the translocation of TR4 from the nucleus to the cytoplasm. Scale bar, 20 μm. C, Translocation of endogenous TR4 from the nucleus to the cytoplasm in response to bexarotene. TR4 was stained with the anti‐TR4 antibody, followed by fluorescein isothiocyanate‐conjugated antibody. The nuclei were stained with DAPI. Scale bar, 20 μm. Data are the means ± SEM of three independent experiments. One‐way ANOVA was used to test differences for statistical significance. *P ˂ .05, **P ˂ .01

Considering that TR4 promotes POMC expression by binding directly to the POMC promoter region, we tested the effects of bexarotene on the interaction between TR4 and the POMC promoter through the ChIP assay. As expected, bexarotene markedly reduced the binding of TR4 to the POMC promoter (Figure 5A). A previous study demonstrated that the subcellular distribution of TR4 is probably associated with its transcriptional regulation of POMC.^19^ Therefore, we examined the subcellular localization of TR4 in response to bexarotene stimulation. We transfected the AtT‐20 cells with a GFP‐TR4 fusion construct to display the subcellular location of TR4. Basically, the exogenously transferred TR4 was exclusively localized in the nucleus of approximately 95% of the cells. Upon the addition of bexarotene, GFP‐TR4 shifted to the cytoplasm of approximately 60% of the cells (Figure 5B). However, this nucleocytoplasmic translocation of TR4 was blocked by a specific nuclear protein export inhibitor leptomycin B (LMB) (Figure 5B). The effect of bexarotene on the subcellular distribution of endogenous TR4 was reflected by the immunofluorescence assay. The endogenous TR4 was localized in both the nucleus and the cytoplasm. The addition of bexarotene induced the evacuation of endogenous TR4 from the nucleus of 60% of the cells (Figure 5C). Overall, results from Figure 5A‐C suggest that bexarotene can inhibit the TR4 function *via* altering the nuclear‐cytoplasmic distribution of TR4.

### 3.6 Preclinical studies using in vivo mouse model to prove the effects of bexarotene to suppress CD

To mimic the clinical drug treatment of CD patients, we tested the efficacy of bexarotene treatment on the growth of existing experimental ACTH‐secreting tumours in vivo. Specifically, the AtT‐20 cells were inoculated subcutaneously in athymic nude mice, and tumours were allowed to develop. After 2 weeks, most of the mice developed palpable tumours, and common symptoms of human CD were observed, such as thinning of skin and fat accumulation around the neck. The mice received either oral bexarotene (100 mg/kg/d) or vehicle for 18 days in random. Tumour volume was monitored every three days throughout the treatment.

**FIGURE 6:**
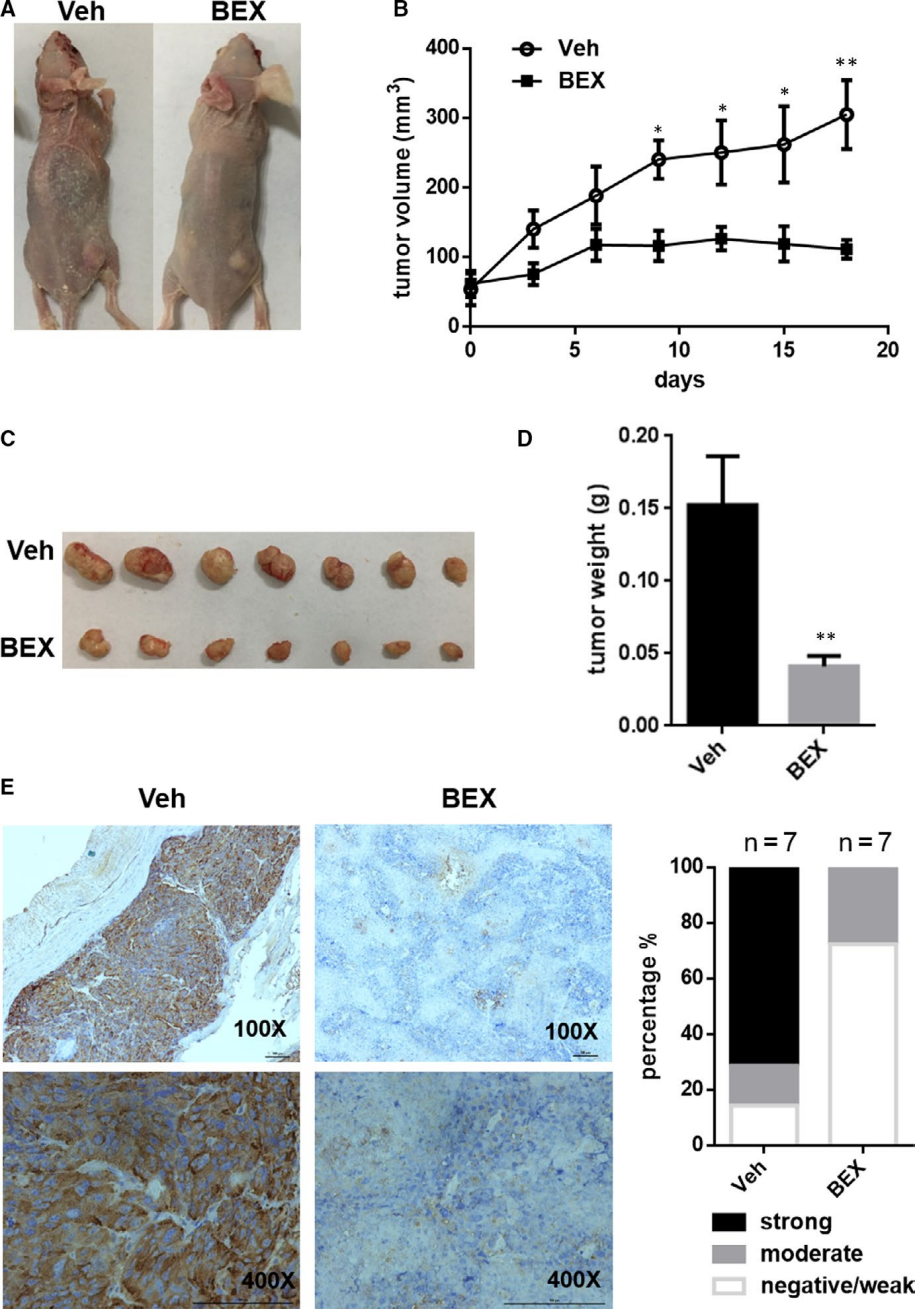
Bexarotene inhibited AtT‐20 tumour growth. A, Representative examples of the mice treatment with bexarotene (100 mg/kg) or vehicle for 18 days. B, Mice with tumours were orally administered with bexarotene (n = 7) or vehicle (n = 7) daily for 18 days. Tumour volume was monitored every three days throughout this period. C, Xenograft tumours of various sizes. D, Tumour weight of bexarotene and vehicle group was measured. Two‐way ANOVA and Student's *t* test were used to test differences for statistical significance of tumour volume and tumour weight respectively. *P ˂ .05, **P ˂ .01. E, IHC was carried out to reveal the expression of POMC protein in the tumour samples of bexarotene‐ and vehicle‐treated group. IHC staining was performed using the POMC antibody (1:400). Left panels show imaging, while right panels show quantification. Scale bar, 100 µm

**FIGURE 7:**
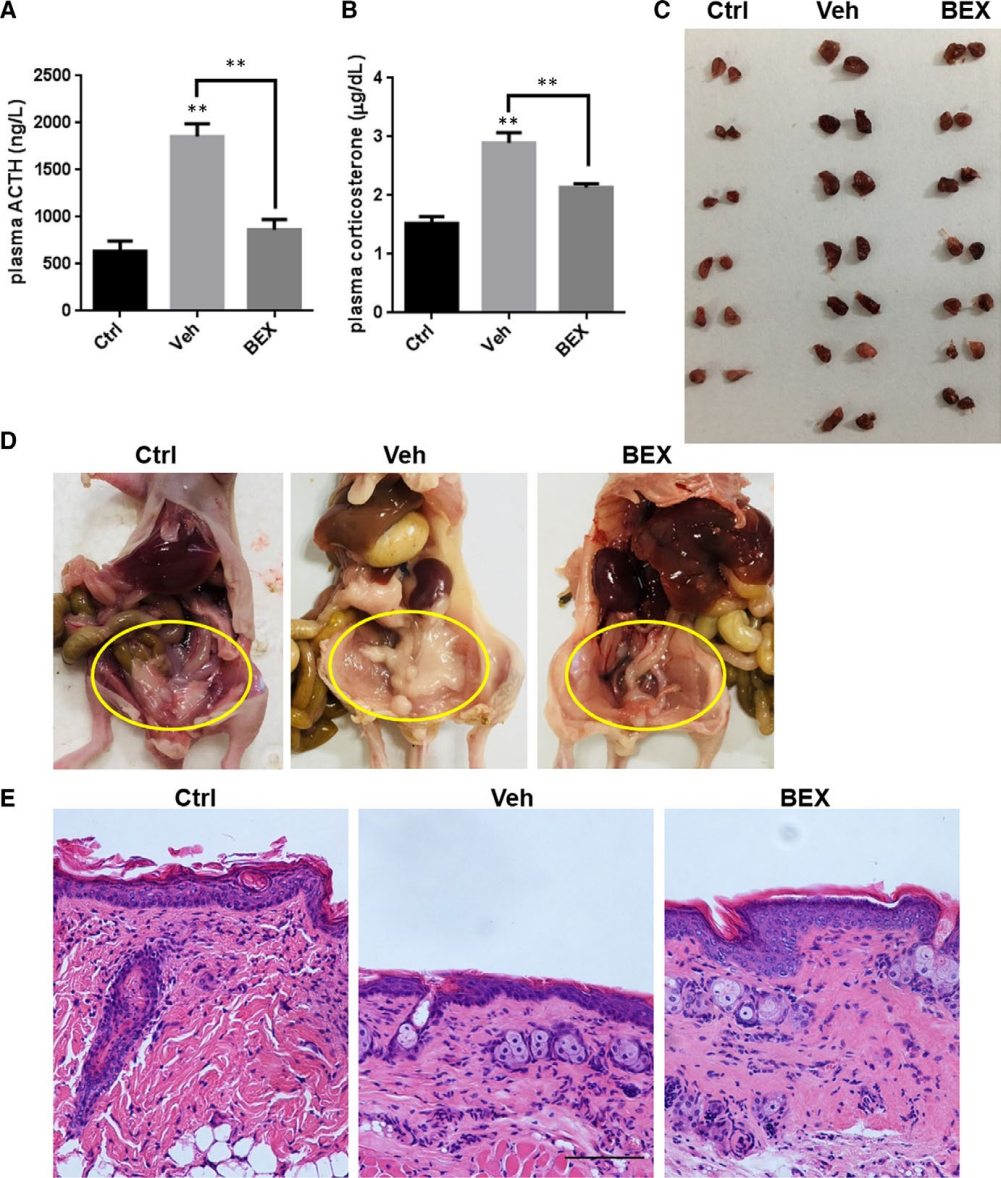
Bexarotene relieved the symptoms of CD. Plasma concentrations of ACTH (A) and corticosterone (B) 24 h after the last treatment. Data are the means ± SEM of three independent experiments. One‐way ANOVA was used to test differences for statistical significance. C, Adrenal gland from normal mice and tumour‐bearing mice treated with bexarotene and vehicle. D, Abdomen fat amount in normal mice and tumour‐bearing mice treated with bexarotene and vehicle. E, Skin samples of normal mice and tumour‐bearing mice treated with bexarotene and vehicle as determined by H&E staining. Scale bar, 100 µm

Notably, bexarotene nearly blocked the growth of ACTH‐secreting tumours (Figure 6A,B), and the tumour weight in the bexarotene group was significantly less than that in the vehicle‐treated group (Figure 6C,D). The results of IHC staining revealed that POMC, CDK1 expression were distinctly reduced while the cleaved caspase‐3 was markedly increased in the bexarotene‐treated xenograft tumours, which is consistent with the in vitro results mentioned above (Figure 6E and Figure S5). Moreover, translocation of TR4 from the nucleus to the cytoplasm by bexarotene administration in the xenograft was further verified via IHC staining (Figure S5). Besides tumour growth inhibition, plasma ACTH and corticosterone secretion were partially but significantly reduced by bexarotene (Figure 7A,B). Other common symptoms of human CD were also markedly relived in mice administered with bexarotene as manifested by the reduced sized of adrenal gland (Figure 7C), diminished fat accumulation around the abdomen (Figure 7D), and decreased thinning of skin (Figure 7E). Finally, no significant differences on cell growth, POMC expression and ACTH secretion were observed in the rat primary pituitary cells upon bexarotene treatment (Figure S6), suggesting bexarotene inhibition effect is specific to ACTH‐secreting adenomas.

Together, results from Figures 6 and 7 indicate that bexarotene alleviates the symptoms of CD in the mouse model.

## 4 DISCUSSION

Although surgical resection offers a good remission rate for the majority of CD patients, only a few drug options were left for patients with inoperable tumours or recurrent diseases. Close biochemical and clinical supervision by an experienced endocrinologist are required for each of the existing drugs due to their adverse effects.^32^ Thus, searching effective and safe drugs for CD treatment is of great importance. In this study, we identified bexarotene as a potential inhibitor of TR4 and demonstrated its potential application in CD treatment. The binding of bexarotene to TR4‐LBD induced a conformation change in TR4 secondary structure and promoted the translocation of TR4 from the nucleus to the cytoplasm, resulting in reduced transcription of POMC.

Bexarotene is a selective RXRs agonist initially approved by the FDA to treat all stages of CTCL. Several clinical trials of bexarotene in the treatment of lung cancer, acute myeloid leukaemia, breast cancer, thyroid cancer, and Kaposi sarcoma are under way, given its curative potency in various cancer types.^33^ In previous clinical practice, the long‐term administration of bexarotene induces the reduction of plasma cortisol levels in CTCL patients in some cases,^23^, ^24^ suggesting its potential for CD treatment. A clinical trial has been launched to investigate the tolerability and effect of a preoperative five‐day course of bexarotene in CD patients before transsphenoidal surgery. However, the effect and mechanism of bexarotene in CD has not been studied. In this study, we found that bexarotene inhibits the cell growth of ACTH‐secreting cells, where both apoptosis and cell cycle arrest are associated with the bexarotene‐induced cell growth inhibition of AtT‐20. The effects of bexarotene on cell apoptosis and cell cycle arrest were also observed under CTCL treatment.^34^, ^35^ Nieto‐Rementeria et al demonstrated that G1/S and G2/M checkpoints contribute to cell cycle arrest induced by bexarotene.^35^ In AtT‐20 cells, we observed that G2 arrest was enhanced with increased bexarotene concentration, whereas the G1 phase was not affected, suggesting the major role of G2 arrest in the bexarotene‐mediated cell cycle arrest. Cyclin D1 serves as the major target of bexarotene in several cancer types, such as colon,^36^, ^37^ lung,^38^, ^39^ and breast cancer.^40^, ^41^ However, we observed that CDK1, which participated in G2/M transition, was dramatically reduced by bexarotene exposure.

Moreover, bexarotene reduced the expression of POMC at the mRNA and protein levels, resulting in reduced secretion of ACTH. In particular, luciferase activity driven by the POMC promoter as inhibited by bexarotene was augmented in the presence of high TR4 level, while this effect was almost abolished by TR4 knockdown. These results suggest that TR4 mediates the effect of bexarotene on AtT‐20. The mRNA and protein amounts of TR4 were not affected by bexarotene exposure, indicating that bexarotene suppressed the transactivation activity of TR4 to reduce its downstream POMC expression.

A previous study demonstrates that TR4 is mainly located in the nucleus of corticotroph tumours, whereas TR4 was not expressed in the nucleus of normal pituitary gland.^19^ The GFP‐tagged TR4 was exclusively localized in the nuclei of the AtT‐20 cells. Treatment with bexarotene promoted TR4 translocation from the nucleus to the cytoplasm. This phenomenon indicates that the nuclear localization of TR4 plays a pivotal role in the transcriptional regulation of POMC. We further performed an immunoprecipitation combined with LC‐MS/MS assay to profile the TR4 binding protein when cells were treated with and without bexarotene. In total, 50 proteins (The keratin protein was excluded.) were identified to bind TR4, 30 proteins were found in both groups, while 10 proteins were exclusively presented in one group (Figure S7). Notably, several proteins belonging to the heat shock protein 70 family (HSP70) were more abundant in the bexarotene group, among which the heat shock 70 kD protein 1A was present exclusively in the bexarotene group. HSP70 has been reported to interact with steroid receptor and helps to stabilize the steroid receptor. The stabilization of steroid receptors deprives them of their capacity to enter the nucleus and initiate their nuclear function.^42^ These data indicated that the addition of bexarotene may promote the sequestration of TR4 in the cytoplasm by chaperones of HSP70 family. Future studies are needed to determine the function and mechanism of bexarotene in regulating the interaction between TR4 and HSP70.

Several attempts have been made for the application of existing clinical drugs in CD treatment. The PPARγ agonist rosiglitazone, an approved drug for type 2 diabetes, exerts inhibitory effects on pituitary tumour growth, tumour ACTH synthesis, and secretion.^43^ Silibinin, an adjuvant drug for hepatitis, can relieve CD in ACTH‐secreting cells and mouse allograft models by targeting heat shock protein 90.^44^ In this paper, we exhibited the drug repurposing of bexarotene for CD treatment by targeting TR4. Recently, a MEK/ERK pathway inhibitor, MEK162, has been tested to treat CD by targeting TR4, because it inhibits TR4 via phosphorylation regulation. In this study, we compared the inhibitory activities of the compounds on ACTH secretion, where 10 µmol/L bexarotene inhibited ACTH secretion by approximately 20%, which is equivalent to 40 µmol/L MEK162. Another advantage of bexarotene over MEK162 is that bexarotene has been clinically applied, and its side effects can be controlled. Bexarotene can inhibit ACTH secretion as stimulated by CRH and strengthen the inhibition of ACTH secretion by DEX. Considering that TR4 alleviates the transcriptional repression of POMC as mediated by glucocorticoid receptor, it may still promote the glucocorticoid sensitivity of CD.

Aside from the suppression of TR4‐mediated signalling, bexarotene treatment may also involve other mechanisms. The role of epidermal growth factor receptor (EGFR) pathway has been implicated in CD pathogenesis, and EGFR has emerged as a potential therapeutic target for CD.^45^, ^46^, ^47^ Bexarotene induced the dose‐dependent repression of EGFR and phospho‐EGFR expression in several lung cancer cell lines.^48^ Lately, another RXR‐specific agonist HX630 was found to exert an inhibitory effect on cell proliferation and ACTH secretion.^49^ Mechanism dissection reveals that HX630 reduced the recruitment of Nur77/Nurr1 heterodimer to the POMC promoter region through the suppression of Nur77 and Nurr1 expression, thus repressed the POMC expression at transcriptional level. Bexarotene may also be involved in the suppression of EGFR‐ and Nur77/Nurr1‐mediated pathways.

In summary, our work demonstrates that bexarotene is a potential inhibitor for TR4. Importantly, bexarotene may represent a new drug candidate to treat CD. Its availability offers an opportunity to reverse several TR4‐mediated pathways involving tumour metastasis and drug resistance.

## CONFLICT OF INTEREST

There are no conflicts of interest for all authors.

## AUTHOR CONTRIBUTIONS

**liqun Xia:** Conceptualization (lead); data curation (equal); formal analysis (equal); funding acquisition (lead); project administration (equal). **Danyang Shen:** Data curation (equal); formal analysis (equal). **Youyun Zhang:** Data curation (equal); formal analysis (supporting). **Jieyang Lu:** Data curation (supporting); formal analysis (equal); methodology (supporting). **Mingchao Wang:** Data curation (supporting); formal analysis (supporting); methodology (supporting). **Huan Wang:** Data curation (supporting); formal analysis (supporting); methodology (supporting). **Yuanlei Chen:** Data curation (supporting); formal analysis (supporting). **Dingwei Xue:** Data curation (supporting); formal analysis (supporting). **Dajiang Xie:** Data curation (supporting); formal analysis (supporting); investigation (supporting). **Gonghui Li:** Conceptualization (equal); project administration (lead).

## Supporting information

Supplementary Material

## Data Availability

The data that support the findings of this study are openly available.
