# Supplementary material for: Targeting the TR4 nuclear receptor with antagonist bexarotene can suppress the proopiomelanocortin signalling in AtT‐20 cells
Source: J Cell Mol Med. 2021 Jan 24;25(5):2404–17. doi: 10.1111/jcmm.16074 (PMC7933964; doi:10.1111/jcmm.16074)
Supplement: Supplementary file 1 — Supplementary Material [file JCMM-25-2404-s001.doc]

Supplementary information

Table S1 PCR primers used in this study

| name | Primer sequence |
| --- | --- |
| actin | forward: 5'-GGCTGTATTCCCCTCCATCG-3' |
|  | reverse: 5'-CCAGTTGGTAACAATGCCATGT-3' |
| TR4 | forward: 5'-GACTCTGCGGTAGCCTCAC-3' |
|  | reverse: 5'-AGGATGAACTGCTGTTTAGAGGA-3' |
| POMC | forward: 5'-CATAGATGTGTGGAGCTGGTG-3' |
|  | reverse: 5'-CATCTCCGTTGCCAGGAAACAC-3' |
| EGFR | forward: GCCATCTGGGCCAAAGATACC |
|  | reverse: GTCTTCGCATGAATAGGCCAAT |
| RARα | forward: ATGTACGAGAGTGTGGAAGTCG |
|  | reverse: ACAGGCCCGGTTCTGGTTA |
| RARβ | forward: GCAGTGCGTGGACACATGA |
|  | reverse: GGCAGGGAGAGTCCTCTGAT |
| RARγ | forward: GGAGCAGGCTTCCCATTCG |
|  | reverse: CATGGCTTATAGACCCGAGGA |
| RXRα | forward: ATGGACACCAAACATTTCCTGC |
|  | reverse: CCAGTGGAGAGCCGATTCC |
| RXRβ | forward: CCACCTCTTACCCCTTCAGC |
|  | reverse: TGGAAGAACTGATGACTGGGA |
| RXRγ | forward: CATGAGCCCTTCAGTAGCCTT |
|  | reverse: CGGAGAGCCAAGAGCATTGAG |

Table S2 siRNAs used in this study

| name | Primer sequence |
| --- | --- |
| siEGFR1# | TCCAACTATGGGACAAACA |
| siEGFR2# | AGTATGCAGATGCCAATAA |
| siRARα | GACACTCTAAGCGGACAGT |
| siRARβ | GCTGGAGAATTCTGAAGGA |
| siRARγ | CCAAGGAAGCTGTAAGGAA |
| siRXRα+β | TGGAGCACCTGTTCTTCTT |
| siRXRα+γ | CATGGGCATGAAGCGGGAA |


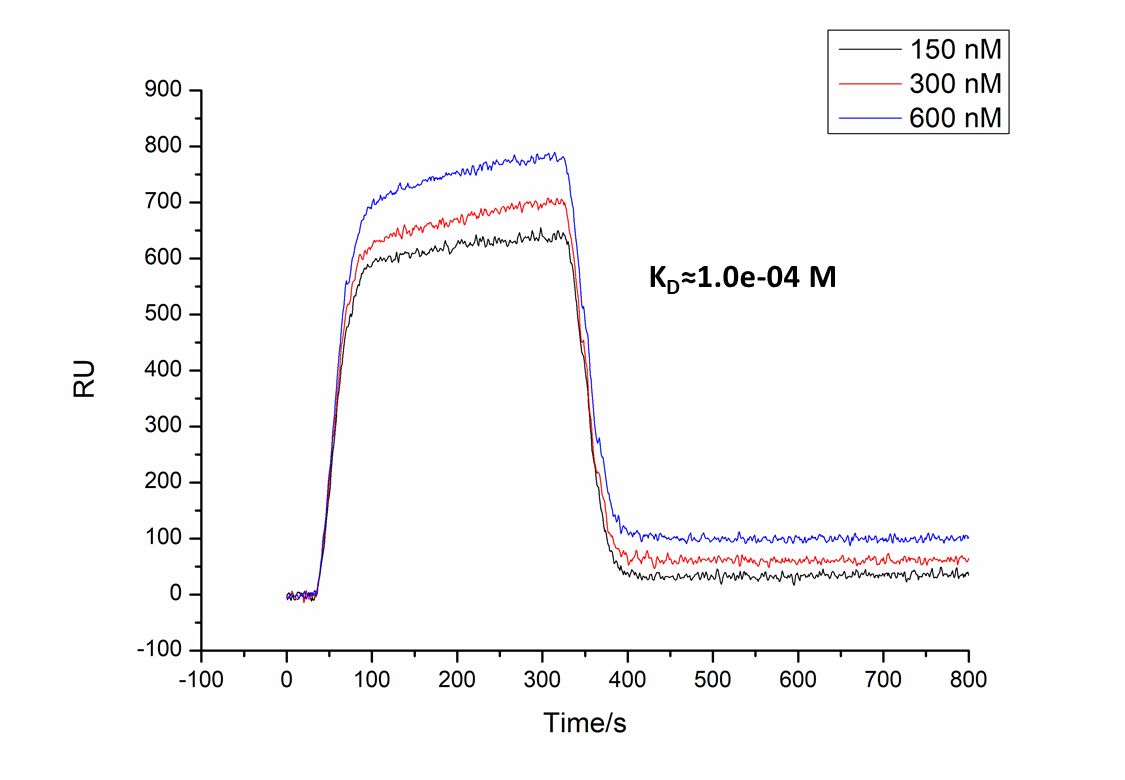


Figure S1. Binding kinetic analysis of ATRA to TR4–LBD by SPR


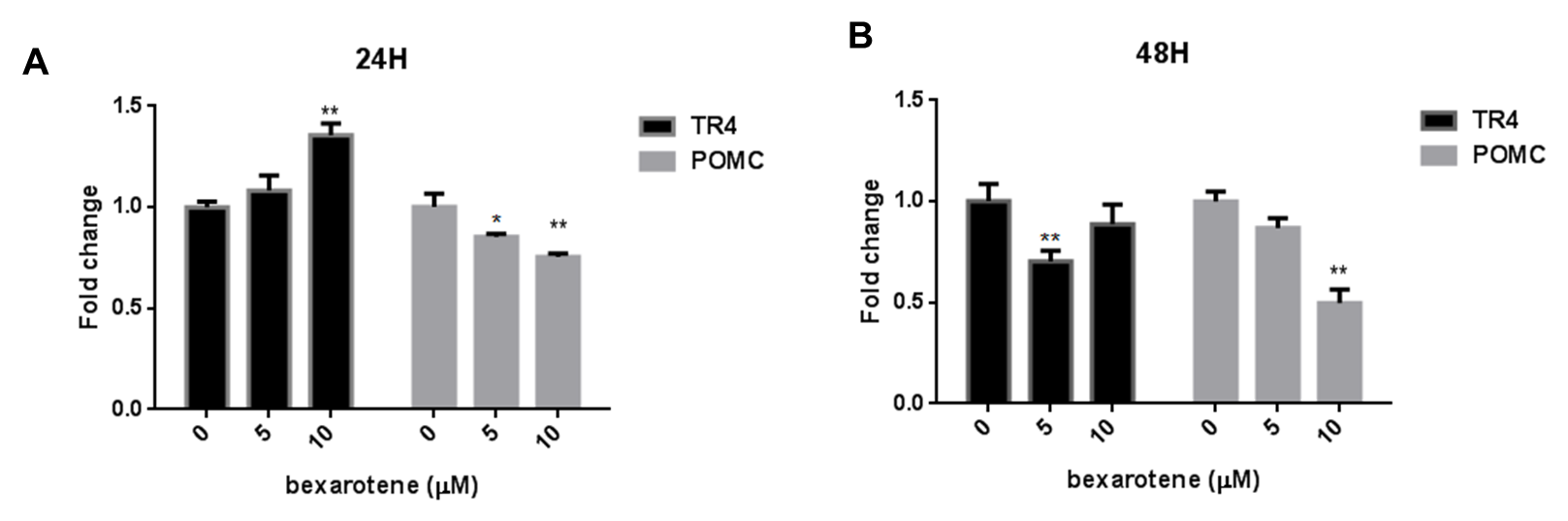


Figure S2. Changes in POMC and TR4 mRNA after treatment with 0, 5 and 10 µM bexarotene for 24 h (A) and 48 h (B) were assessed by qPCR. Data are the means ± S.D. of three independent experiment. *P ˂ 0.05, **P ˂ 0.01. One-way anova was used to test differences for statistical significance.


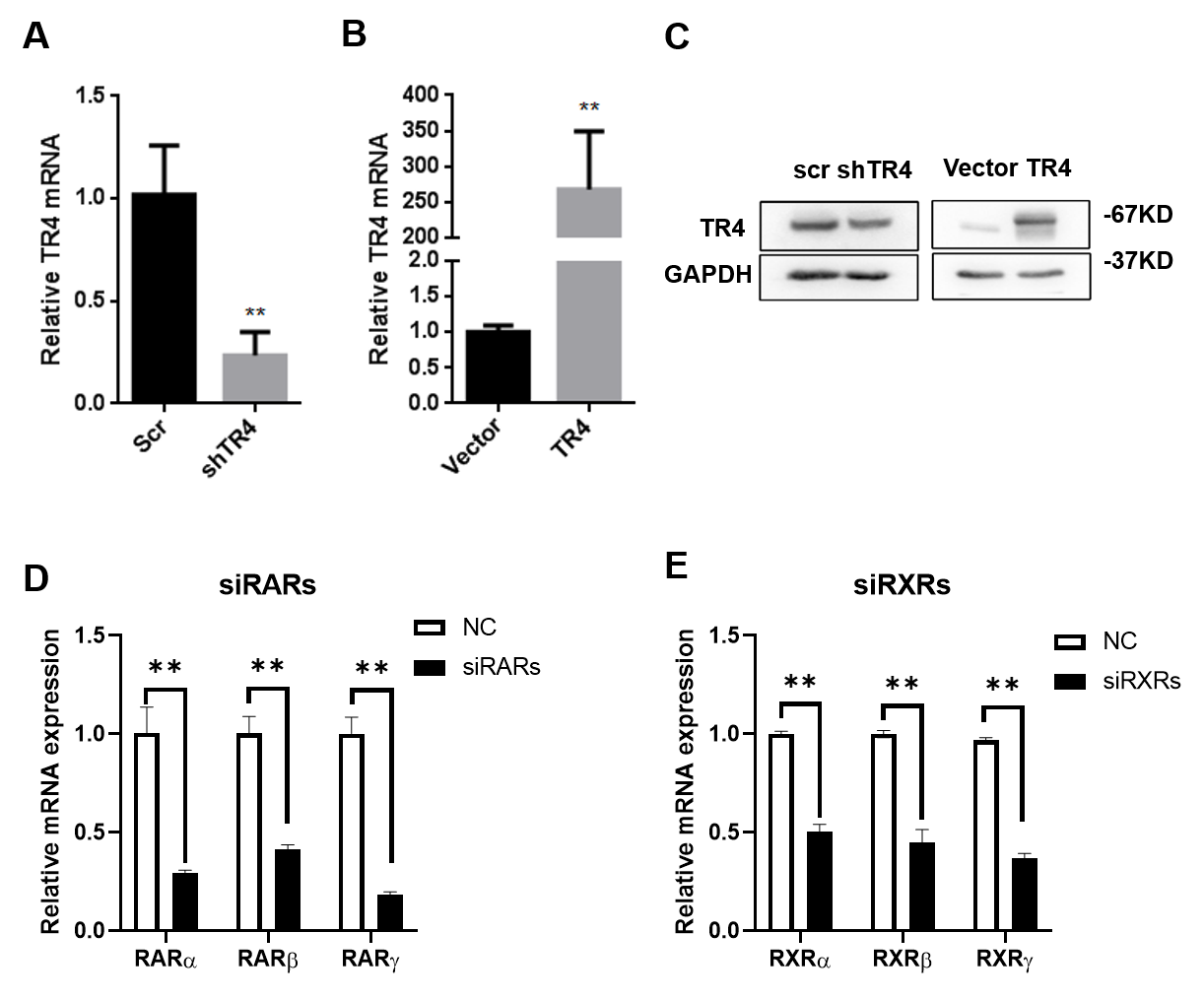


Figure S3. A, Knockdown efficiency of shTR4 plasmid was evaluated by qPCR; B, Ectopic expression of TR4 in AtT-20 cells was confirmed through qPCR; C, Western blot analysis to confirm knockdown and overexpression of TR4; D, Knocking down of RARα/β/γ by siRNA; E, Knocking down of RXRα/β/γ by siRNA. Data are the means ± S.D. of three independent experiment. *P ˂ 0.05, **P ˂ 0.01. One-way anova was used to test differences for statistical significance.


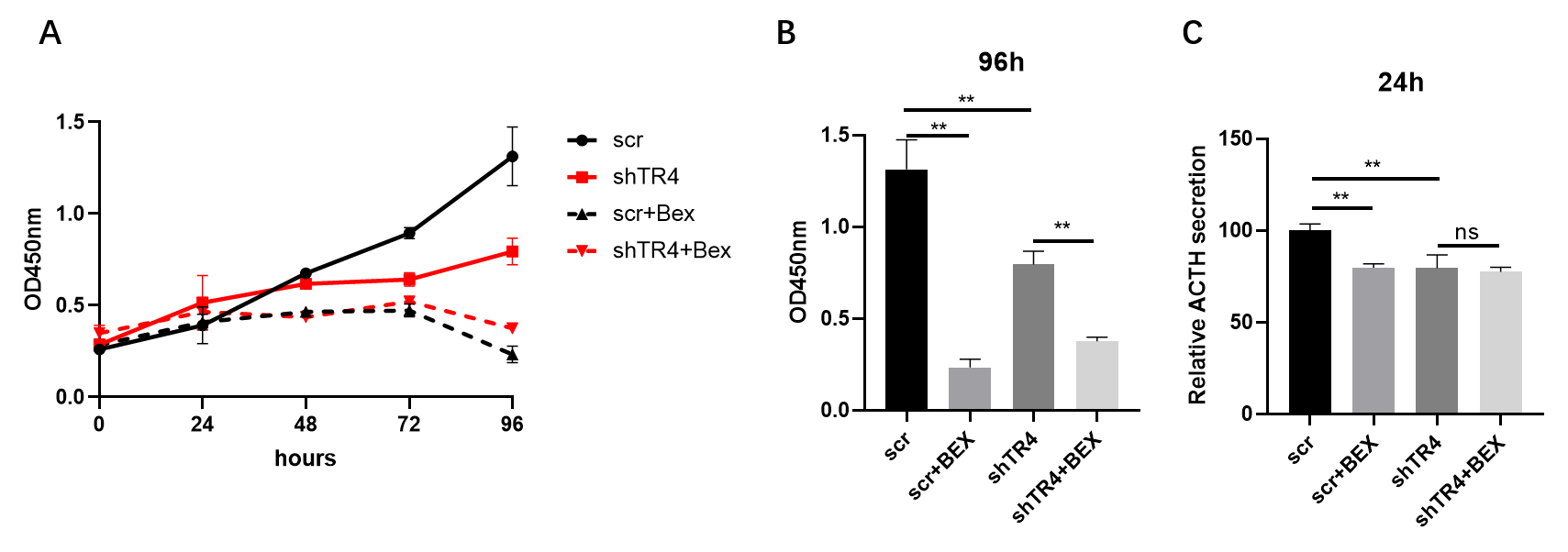


Figure S4. A, proliferation of scr/shTR4 AtT-20 cells treated with or without bexarotene (10 µM) for 0, 24, 48, 72, 96 h was measured with CCK8 assay; B, Survival of scr/shTR4 AtT-20 cells treated with or without bexarotene (10 µM) for 96 h; C, ACTH secretion of scr/shTR4 AtT-20 cells treated with or without bexarotene (10 µM) for 24 h was measured with ECLIA assay. Data are the means ± S.D. of three independent experiment. *P ˂ 0.05, **P ˂ 0.01. One-way anova was used to test differences for statistical significance.


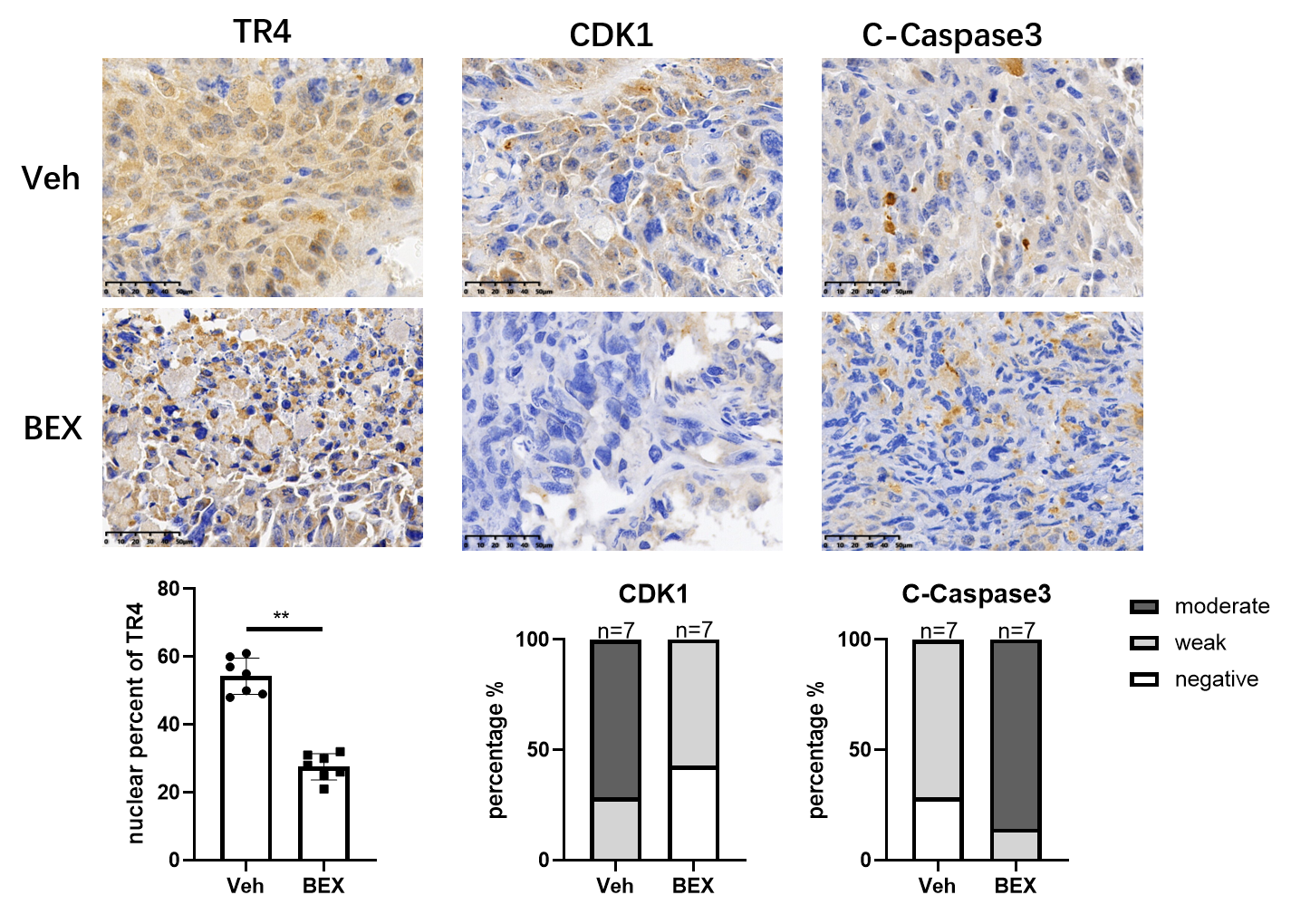


Figure S5. IHC analysis of TR4, CDK1 and cleaved-caspase in pituitary tumors treated with or without bexarotene.


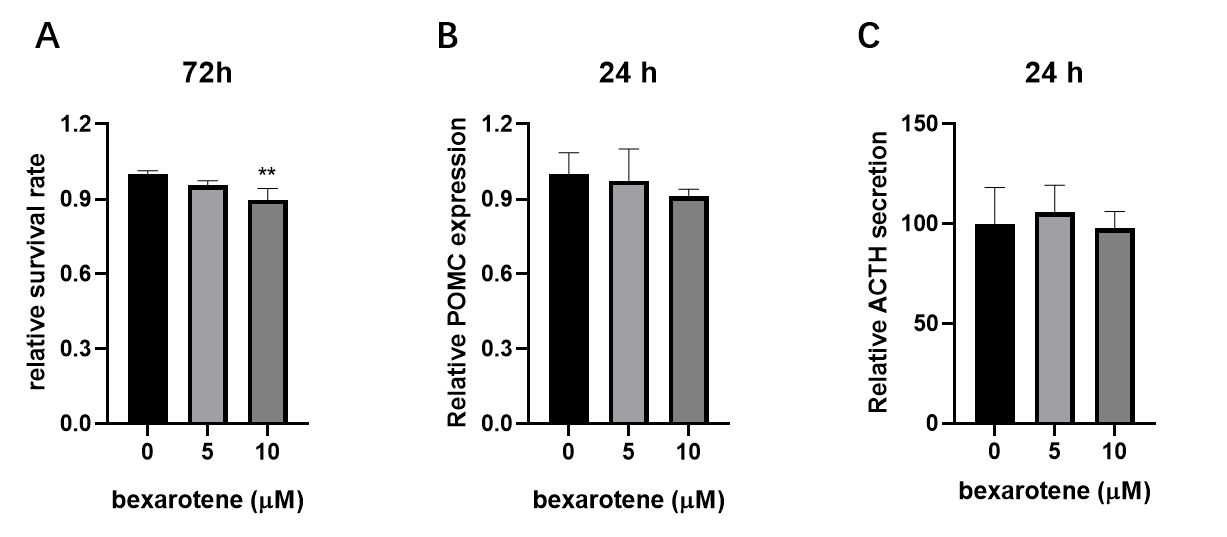


Figure S6. A, proliferation of primary pituitary cells treated with 0, 5, 10 µM bexarotene for 72 h was measured with CCK8 assay; B, qPCR analysis of POMC in primary pituitary cells treated with 0, 5, 10 µM bexarotene for 24 h; C, ACTH secretion of primary pituitary cells treated with 0, 5, 10 µM bexarotene for 24 h was measured with ECLIA assay. Data are the means ± S.D. of three independent experiment. *P ˂ 0.05, **P ˂ 0.01. One-way anova was used to test differences for statistical significance.


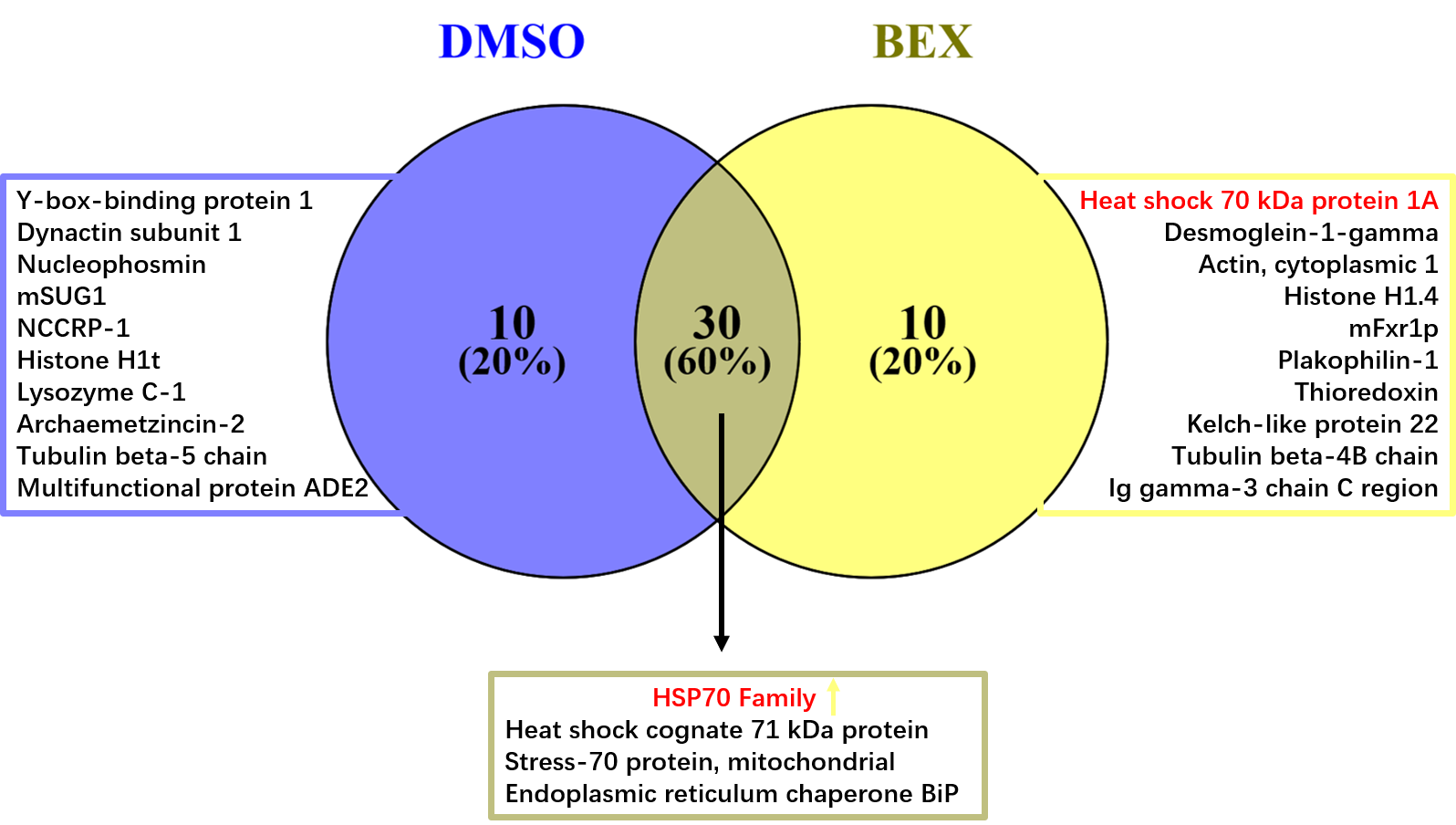


Figure S7. Proteins interacted with TR4 when AtT-20 cells were treated with or without bexarotene. The 10 proteins in the blue box were exclusively present in the DMSO group, while the 10 proteins in the yellow box were exclusively present in the BEX group. The 3 proteins belonging to the HSP70 family were more abundant in the BEX group.

**TR4 immunoprecipitation and LC-MS/MS analysis:**

AtT-20 cells incubated with or without bexarotene for 24 h were harvested and lysed in binding buffer. Subsequently, the protein concentration was determined by BCA assay. The same amount of protein extracts from both group were then incubated with Anti-TR4 antibody overnight at 4℃ with agitation. Then agarose resin were added and incubated for 3 h at 4℃ with agitation. The resin was then washed five times with binding buffer. The pulled-down protein complexes were then examined by LC-MS/MS.
